# “There Needs To Be More People Sharing That Information Across the Nation”: Opportunities to Promote HIV Pre-Exposure Prophylaxis Awareness and Uptake among People Releasing from Jail or Prison

**DOI:** 10.21203/rs.3.rs-10182163/v1

**Published:** 2026-07-16

**Authors:** Susan M. Graham, Helene Starks, Emily J. Callen, Eamonn J. McGonigle, Gina Liu, Zoe Senter, Keonna Moffett, Winter Forsyth, Brett Hauber, Liying Wang, Courtney Bagdon-Cox, Helen E. Jack

**Affiliations:** University of Washington; University of Washington; University of Washington; University of Washington; University of Washington; University of Washington; Clinical Social Worker Private Practice; University of Washington; University of Washington; Florida State University; Washington State Department of Corrections; University of Washington

**Keywords:** HIV, pre-exposure prophylaxis, motivation, jail, prison

## Abstract

People releasing from jail or prison are at increased risk for contracting HIV, especially in the post-release period, yet there is low uptake of pre-exposure prophylaxis (PrEP). To investigate the potential for increasing PrEP use in this population, we conducted 15 semi-structured interviews with individuals who were released from a Washington State jail or prison between 2018–2023. Participants were recruited with help from community-based organizations focused on HIV prevention, behavioral health, or post-release service provision. Many participants perceived themselves to be at risk for HIV, several were aware of PrEP, and a few were interested in taking PrEP. Most, however, faced structural barriers to PrEP access and felt that they needed more information about HIV risk and prevention before deciding whether PrEP was right for them, particularly in light of competing priorities. Efforts to prevent HIV in this population should focus on increasing awareness and knowledge about HIV risk and about PrEP as a prevention tool, both during and after incarceration. If ambivalence about PrEP use is addressed and interest increases, low-barrier access will be critical for those motivated to use PrEP for HIV prevention.

## Introduction

In the United States, release from prison or jail can be an especially high-risk time for HIV acquisition, as many individuals return to behaviors such as injection drug use [1], exchanging sex for drugs or money [2], or engaging in condomless sex with multiple or concurrent partners [3]. Moreover, the majority of people releasing from jail or prison experience behavioral health challenges that further increase HIV risk [4–6]. While estimates vary widely, the prevalence of mental health disorders in U.S. prisons is likely over 50% [7]. In a survey conducted by the American Psychological Association, 64% of people in jails, 54% of people in state prisons, and 45% of people in federal prisons reported anxiety, depression, or serious psychological distress that limited their ability to function [8]. Substance use disorders are also prevalent, as one in five incarcerated people in the U.S. has a drug offense [9], and some continue to use drugs during incarceration [10]. In a recent study of people released from prison in Washington State, where the current research was conducted, the estimated risk of HIV acquisition was 6.1 times higher than the rate among individuals not released from prison, with the highest risks found among those with substance use disorders, mental health conditions, and low educational attainment [11].

Many people releasing from jail or prison could benefit from HIV preexposure prophylaxis (PrEP). In a study of women on probation, parole, or recently released from jail or prison in Connecticut, one third were considered candidates for HIV PrEP [12]. In a prospective cohort of men incarcerated in the Rhode Island Department of Corrections system, 83% were determined to be eligible for PrEP based on established HIV risk criteria [13]. Despite the substantial proportions of people in jail or prison who meet PrEP eligibility criteria, uptake of HIV PrEP in people releasing from United States jails and prisons has been limited to date [14]. The Rhode Island Department of Correction system’s PrEP program provides the most comprehensive data to date among persons releasing from prison or jail and reports that only 15%−26% of individuals who were offered PrEP initiated use, and just 5% were successfully linked to PrEP care in the community [13, 15].

While competing health and social priorities post-release, structural barriers to medication access such as lack of insurance and poor access to transportation, and challenges due to ongoing substance use have been noted as key barriers to PrEP engagement in prior qualitative studies [16–18], low knowledge about HIV transmission and PrEP has been identified as an important barrier to HIV prevention behavior change among criminal-legal-involved individuals [12, 16, 19]. Lack of knowledge contributes to post-release HIV acquisition through underestimation of personal risk and limited awareness of prevention options [12, 20]. Both PrEP awareness and PrEP knowledge are often limited among people with criminal legal involvement, yet interest in PrEP increases after basic education about this HIV prevention tool [14, 16, 18, 21, 22]. Unfortunately, opportunities to learn about PrEP within corrections are limited [12, 18, 20], and basic education about HIV and PrEP may not be enough to address underestimation of risk and low motivation [20].

The motivational PrEP cascade, first described in 2017 [23], offers a conceptual model of PrEP uptake as a series of sequential stages based on an individual’s readiness and motivation to use PrEP. The motivational PrEP cascade differs from the traditional PrEP care continuum, which focuses on operational steps in PrEP delivery (i.e., identifying at-risk individuals, facilitating access, linking to care, prescribing, promoting adherence, and retaining in care) from a provider perspective [24]. Specifically, the motivational cascade emphasizes the psychological readiness and behavioral stages individuals at risk for HIV acquisition progress through, making it particularly useful for understanding barriers and designing targeted interventions for populations at different stages of readiness [25, 26].

Guided by the motivational PrEP cascade, our goal in this analysis was to understand how individuals releasing from jail or prison think about their HIV risk, how PrEP and other HIV prevention strategies fit into their priorities, and how low-cost interventions might be used to promote contemplation of PrEP and prepare people who are motivated to use PrEP to engage in HIV prevention services before and/or after release from jail or prison.

## Methods

### Study design.

This qualitative study is reported according to the Consolidated Criteria for Reporting Qualitative Research (COREQ) checklist [27]. We conducted semi-structured, in-depth interviews at a single point in time with three stakeholder groups: 1) people released from jail or prison (i.e., people with lived experience [PWLE] of incarceration) between 2018–2023; 2) prison- and jail-based clinical and reentry planning staff; and 3) clinical and other program staff within community-based organizations (CBOs) focused on HIV prevention, behavioral health, or post-release services. The current analysis focuses on HIV risk perception and PrEP uptake among PWLE. Findings from staff interviews [28] and content from PWLE not focused on PrEP (Forsyth, in submission) are presented elsewhere.

### Study team and reflexivity.

Our authorship team includes a diversity of perspectives as researchers in HIV prevention, implementation science, criminology, and health economics and clinicians in internal medicine and infectious diseases. Team members are based in academic institutions and the Washington Department of Corrections, allowing us to ground our implementation research in partnership with an implementing institution.

### Sampling and recruitment.

We asked our CBO staff participants to help recruit a diverse group of participants released from prison or jail in Washington State between 2018–2023. These staff asked potentially eligible participants for permission to share contact information with our study team who then did outreach by phone or e-mail. Some recruitment was also done in person, outreaching directly to people accessing care at CBO offices, to overcome barriers due to limited phone or computer access. Participants were eligible if they had not previously tested positive for HIV and had been released from jail or prison within the previous 5 years at the time of the interview. We reached our *a priori* goal of 15 participants after 6 months of recruitment (May through October 2023).

### Data collection.

All study procedures took place with only the research staff and participant present at a meeting by telephone, HIPAA-compliant Zoom, or in person in a private space. After providing informed consent, participants completed a brief survey to report on their sociodemographic information, date of release, and whether their most recent release was from jail or from prison. For the semi-structured interviews, we used a life history approach to elicit stories about experiences after incarceration and provide insights into participants’ lives and broader social contexts [29]. PWLE were also asked about their priorities upon release, any health concerns, and thoughts about HIV risk or HIV prevention after incarceration. This included a discussion of whether participants had heard about PrEP and what they thought about taking “a daily pill or an injection given every other month that prevents people from getting HIV.” All interviews were audio recorded, transcribed using Otter.ai software, and checked and corrected as needed. The topic guide used is presented in Supplemental Material 1.

### Data analysis.

Participant demographics were summarized using descriptive statistics in Stata version 14.0. HEJ conducted one interview in Spanish; this interview was professionally translated after transcription. As part of a broader qualitative analysis, we focused on identifying themes related to HIV risk and PrEP awareness and perceptions [30, 31]. Our initial codebook deductively identified content on HIV risk perception, responses to PrEP-related interview guide questions, and domains in the behavior change wheel (i.e., the COM-B framework). COM-B posits that Capability (i.e., physical or psychological ability to change behavior), Opportunity (i.e., external physical or social factors constraining behavior change), and Motivation (i.e., internal mental processes that underpin desire or willingness to change behavior) are all necessary components for a Behavior to occur [32].

As we applied the deductive codes, we modified labels to align with participant language (“in vivo” codes) and created additional codes for new concepts that emerged inductively; previously coded transcripts were reviewed for use of these codes and updated as needed. Each transcript was coded by two of five investigators on the coding team (EM, GL, ZS, WF, EC), and differences in code assignments were reconciled through discussion. After this initial coding was complete, we analyzed themes within and across cases through multiple rounds of coding and reflection to identify patterns [31].

For this manuscript, SMG closely reviewed the portions of each coded transcript related to HIV risk perception and PrEP to identify COM-B themes and the context in which they emerged. Patterns of thinking that emerged from our data about motivation, a key COM-B construct, fit within the well-established Transtheoretical Model of Prochaska and DiClemente [33, 34] and the motivational PrEP cascade based on this model that was published in 2017 [23]. We therefore used the motivational PrEP cascade steps (i.e., precontemplation, contemplation, preparation, action and maintenance) as a framework to present major themes related to motivation to use PrEP. As very few of our participants were actually taking PrEP, we collapsed the last two motivational PrEP cascade stages related to action and maintenance. Member checking was not conducted.

## Results

Table 1 presents the sociodemographic characteristics of the 15 participants. Overall, the sample was diverse with respect to age, sex, gender identity, and racial/ethnic background. Most participants (80%) were released from jail rather than prison. Educational attainment was limited, with half having a high school diploma or equivalent credential (n = 4) or less than high school education (n = 4). Ten of the 15 participants talked about personal substance use in their interviews, and all mentioned challenges with mental health. Eight participants mentioned various types of stigma (e.g., incarceration stigma, substance use stigma) as a challenge and 10 mentioned past trauma in their interviews.

**Figure 1:**
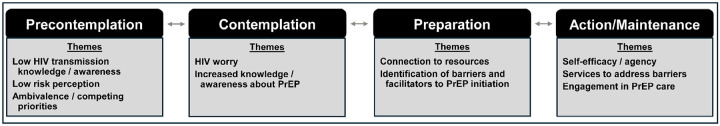
Stages in the motivational PrEP cascade with key themes related to HIV prevention and PrEP use related to each stage. Movement between stages can be forward or backward, depending on circumstances. Support for each stage should happen both in carceral settings and in the community after release.

We summarize the overarching themes from interviews below. The first set of themes related to the first two components of the COM-B model: capability and opportunity. To explore motivation, the third component of COM-B, we mapped results according to the motivational PrEP cascade (Fig. 1 below). Additional quotes related to individual experiences and recommendations are presented in Table 2.

### Capability and opportunity to use PrEP.

Participants felt that carceral policies related to abstinence from sex and drugs created barriers to reducing HIV risk and using PrEP, both during incarceration and after release. They recommended increased opportunities for health education, interaction with knowledgeable peers and providers, and linkage to HIV prevention services and resources, ideally in a centralized location. Several participants mentioned that opportunities to learn about HIV prevention and access PrEP were lacking, both in jail and prison settings and in the community:

“I feel like it’s very difficult to get any type of medication that might be viewed by custody as encouraging certain activities like drugs or sex… Custody does not want, you know, the health care professionals prescribing a medication like that or any other medication, because they’re convinced that it will cause drug use.”

‒ Multiracial transgender woman, mid 40s

“When I’m out on the streets doing what I’m doing every day,…I don’t necessarily have the awareness of these programs that are going to help me with these resources…When [the CBO staff] come to me and either bring the entirety of those resources to my location…or bring instructions on how to go and receive them, it is incredibly helpful.”

‒ White cisgender man, late 20s

Limited availability of certain types of care (e.g., gender-affirming care for transgender persons, other specialty care) in jails and prisons sometimes persisted during community custody due to constraints on travel.

“…I can’t leave the county. The county thing has turned out to be a bit of a problem…I had to turn down a medical visit because the provider assigned to me turned out to be just over the county line. And I didn’t know it until after I’d started going that way… And I had to call and cancel the appointment…”

‒ White transgender woman, late 50s

### Motivational PrEP cascade.

Motivation to use PrEP was heavily influenced by HIV risk perception and knowledge about PrEP, especially for individuals in the earlier stages of the PrEP cascade. Figure 1 presents key themes related to the motivational PrEP cascade among study participants by stage of readiness for change. Support for each stage should happen both in carceral settings and in the community after release. The major themes within each stage of readiness for change are described below in the following sections.

Precontemplation. Participants in the precontemplation stage had limited or incorrect knowledge, perceived themselves to be low risk or were ambivalent about HIV prevention and prioritized other things. As a result, these individuals did not self-identify as PrEP candidates or were not open to contemplating PrEP initiation.

“I don’t even know if I believe in it [HIV]. You know, like, I’m sure it was a significantly impactful concern in the past. But this is 2023. I don’t think it’s that much of a thing. If I were significantly more sexually active, and in a community of generally, older people, perhaps I might take it. But I’ve been sexually active with people of ages 40 and under my whole life. I believe that that age range…has got to be a little bit lower risk in general than, like older folks. And also, I’m not having nearly that much sex or involved in nearly that much needle usage…”

‒ White cisgender man, late 20s

“It [HIV] was something I thought about, but it didn’t stop me from doing incredibly dangerous and irresponsible things. Because chances are I’m gonna die from an overdose or the lifestyle before HIV would get me… I know that using, sharing needles is…high risk behaviors. And I know…all this stuff… and I still chose to do it. So what are we talking about? I mean why are you gonna tell me how to prevent it when I’m actively not preventing it?”

‒ White cisgender man, late 30s

Participants thought that hearing about risks from someone they could trust, such as a peer with similar experience, could make people start thinking about the risk of HIV acquisition from sex or substance use practices:

“If we actually had people who knew exactly what they were talking about, who could come in and talk to us, in prison and in jail about the possibility of spreading HIV or STDs, I think people would actually be more careful and more serious in their relationships and in being cautious about what they do.”

‒ Black transgender man, early 30s

Contemplation. Several participants did perceive themselves to be at risk for HIV and indicated that they could potentially see themselves as candidates to take PrEP. For some, specific circumstances drove this interest. Others felt they might be interested if they had more information or if their circumstances changed in the future.

“At the moment, I’m not gonna lie, I have a boyfriend. And up until just recently, last couple of months, I got this gut feeling that there’s something else going on. So I’ve been making him protect himself, because if he is cheating, I don’t want what [anybody] got…. if [PrEP is] available, I would be interested in doing that, because of my lifestyle.”

– White cisgender woman, early 50s

“If I’m in a position where I’m considering sexual activity, yes, absolutely I’d be [motivated to take PrEP]. I’d be motivated to investigate it more thoroughly than just what I see on a commercial… You know, I want to get a wider view of it and get a little better educated about it…The commercials…make it feel like, well, I can do whatever I want as long as you’re on the drug, and I’m not entirely sure that that’s the case.”

–White transgender woman, late 50s

For participants in this stage, knowledge was something they felt would empower people to think about their risk:

“That’s a cool thing [PrEP]…especially in someone that’s in active addiction, that would be a really useful thing… HIV is a serious thing…I think a lot of people are, are not knowledgeable on the situation. And there needs to be more people sharing that information across the nation…Because a lot of people are sharing needles, and they’re so jumped out that they don’t even care if they get HIV. But if they had that availability, they might take that [PrEP], and it might save their life…”

– White cisgender male, early 40s

Participants mentioned several potential means to increase awareness and knowledge for those contemplating whether PrEP would be right for them, including television commercials, social media, apps such as Grindr, and informative posters, fliers, or pamphlets. Participants recommended that PrEP-related information be posted at clinics, pharmacies, CBOs, and government health and social agencies: *“anywhere that touches…a wide scope of people”* or *“in popular places where it’s needed.”*

Preparation. Participants at this stage indicated their intent to start PrEP but had not yet started. They sometimes knew of a medical provider who would prescribe PrEP or a clinic or other place with PrEP services. A few participants mentioned steps they would take to prepare for PrEP initiation. The themes related to this step involved connection to resources and the identification of barriers and facilitators to engagement that would need to be overcome for them to use PrEP. Example quotes are below.

“I took it upon myself to go look for like, HIV PrEP. … The only time I ever hear people openly talk about being on HIV PrEP is when I’m on a website, or when I’m talking with my community, right because…I think it also comes down to… making sure each other is okay.”

‒ Pacific Islander nonbinary person, early 30s

“From what I understand at this point, PrEP is available. And there’s at least one company that’s making it available either at low cost or no cost. A lot of folks either don’t know where to get it or how to get it… I think that would be great if there was some information about how to get access to [PrEP] as part of the release process, along with other resources.”

‒ Multiracial transgender woman, late 40s

In addition, a few participants expressed ways that they or others in their situation could be better prepared for PrEP use. Some shared that access to PrEP while in custody would help:

“I probably would have started it in jail. That probably would have been the jumpstart of me taking the meds.”

– Black cisgender woman, late 30s

In addition to being able to start PrEP before release, participants mentioned several facilitators that would help prepare for PrEP engagement in the community, including referral to a primary provider who prescribes PrEP, free care or insurance coverage for PrEP services, co-provision of PrEP with mental and/or behavioral health services (e.g., medication for opioid use disorder), supportive care without stigma, and addressing concerns about drug interactions or other impacts on health.

Action/maintenance. Only three participants reported that they had engaged with a PrEP provider, started PrEP, or were currently taking PrEP. These participants mentioned help from CBO services as key to addressing barriers to PrEP use and increasing self-efficacy to take PrEP:

“Have a program, like this one [a local CBO that linked him to PrEP after release from jail], which really helps a lot. The people really want to do their job, as I see them here… They don’t mind calling, [helping with paperwork], things like that. Communication more than anything… You just have to remember. I have [a pill box marked with] Monday, Tuesday, Wednesday, Thursday and Friday…and [am] taking it.”

– Hispanic cisgender man, late 30s

One participant recommended that people be able to take PrEP while still in prison or jail and have autonomy to use it discreetly, to avoid stigma and other negative consequences:

“I don’t think it should be a pill-line medication, because I don’t believe that you should have to go to pill-line to get a medication that you take once or twice a day….I believe that you should be able to have it in your room and not have to be criticized for taking care of yourself just in case. People are allowed to have a sex drive and protect themselves, too.”

– Black transgender man, early 30s

When asked what might help others act to initiate PrEP and continue PrEP engagement in the community, participants with some knowledge or experience of PrEP services mentioned several potential facilitators, including reminder tools (e.g., pill boxes), home delivery of medications, telehealth, long-acting PrEP formulations, and supportive providers, partners and family members.

## Discussion

In this qualitative study of people releasing from prison or jail in Washington state, we found that perceptions of HIV risk varied across participants, with many likely underestimating their potential risk in the post-release period. Several were aware of PrEP, and a few were interested in taking PrEP. Most, however, felt that they needed more information about HIV risk and prevention before deciding whether PrEP was right for them. Overcoming low awareness and knowledge about HIV transmission and grounding risk perception in that knowledge is essential in order to increase the motivation of those in the precontemplation and contemplation stages of the motivational PrEP cascade. Participants suggested many ways in which this could be done both during and after incarceration, including increasing access to information and education regarding HIV and discussions with knowledgeable peers and providers. Those who were interested in PrEP felt that access should be given before release if requested, to support HIV prevention as people release. Upon release, participant advocated for facilitated linkage to community resources that provide PrEP information and services, address individual and structural barriers to accessing services, and support sustained service engagement.

Effective interventions to move individuals at risk for HIV forward in the motivational PrEP cascade could leverage psychological factors that may advance motivation, such as worry about health, perception of heightened HIV risk, positive attitudes toward PrEP, and perceived health benefits of PrEP [35]. While the evidence for such interventions targeting people releasing from jail or prison is limited, motivational interviewing (MI)-based interventions have been shown to move individuals forward in the motivational PrEP cascade, with multiple randomized controlled trials demonstrating efficacy of such interventions for improving PrEP uptake and adherence [36–39]. For example, in an trial with men who have sex with men recruited from a sexually transmitted infection clinic, participants receiving a two-session MI intervention targeting low risk perception, stigma, and cost barriers were significantly more likely to attend a PrEP appointment and accept a prescription compared to treatment-as-usual (52.3% vs 27.9%; OR = 3.6) [38]. A similar brief MI intervention for men who have sex with men tripled PrEP prescription rates from 11% to 37% in a small pilot study [39]. Among Black women, a two-session MI-PrEP intervention incorporating psychoeducation and light case management significantly increased the likelihood of speaking to a provider about PrEP (OR = 4.42) and improved PrEP knowledge, motivation, and prescription rates [37]. A scoping review of studies that have used MI interventions to increase PrEP initiation and adherence concluded that interventions that incorporate PrEP education, identify barriers to PrEP use, and develop strategies for overcoming barriers have been the most successful [36]. Research on the adaptation of such interventions for people in jail or prison and on the optimal timing before or after release is needed.

Contingency management (CM) with financial incentives has been successful at improving retention on medications for opioid use disorder among persons releasing from jail [40] and shows promise for increasing PrEP use as well. For example, in a pilot sequential multiple assignment randomized trial among sexual minority men who use stimulants, CM (offering $50 for PrEP evaluation and $50 for filling prescriptions) was more effective than an MI intervention at increasing rates of verified PrEP use over 6 months [41]. Importantly, one intervention tailored to the post-release period has combined motivational and CM components: the Mobile-Enhanced Prevention Support for People Leaving Jail (MEPS) included support from a peer mentor, incentives to engage in social and health services, and a mobile app [42]. This intervention significantly increased PrEP uptake (adjusted odds ratio 3.8) among people transitioning from prison or jail to the community [42]. It would be challenging to use CM interventions within carceral settings, given constraints on payments and rewards. Within such settings, given limited resources, low-cost but well-designed behavioral nudges [43], such as strategically placed posters and fliers, could easily be integrated into health services and educational programs to increase PrEP awareness.

Many of the people recently released from incarceration in our sample faced significant structural barriers to accessing PrEP, including poverty, unemployment, housing and food insecurity, incarceration stigma, and ongoing oversight from the carceral system. These barriers create challenges in accessing healthcare in general and deprioritize preventive services such as PrEP [14, 18, 28, 44]. As such, interventions to increase PrEP awareness and education for this population may fail unless the structural inequities that lead to both greater HIV risk and less PrEP access in this population are also addressed. Wrap-around services that reduce structural barriers are a vital approach to improving social and health outcomes for this population in general [45, 46] and could incorporate opportunities to learn about and access PrEP. Incarceration may provide a space in which housing and food are provided and substance use patterns are disrupted. As one of our participants said: *“It’s tough at first, but I feel like it’s happened for a reason. There’s a reason I got locked up.”* HIV prevention programming and policy should identify effective ways to seize this opportunity when available.

This study had many strengths, including its recruitment of a sample of criminal-legal system-involved individuals who could reflect on their recent release and the barriers to HIV prevention they faced, use of a structured interview format that elicited participant stories prior to focusing on PrEP, and foundation in the COM-B model with expansion to include the motivational PrEP cascade [23, 32]. There were several limitations that should be acknowledged, however. First, our focus was on PrEP awareness and uptake. More information about what it takes to promote long-term PrEP engagement and retention on PrEP among criminal-legal-involved individuals is needed. Second, because we recruited individuals willing to participate in research and who were already connected with a CBO in some way, our sample was biased towards individuals who were more engaged with care and may have been more likely to be contemplating behavior change. Finally, the majority of our participants had been released from jail rather than prison settings and all were from Washington state, potentially limiting the generalizability of our findings to prison settings and beyond Washington state.

In conclusion, efforts to prevent HIV among people releasing from prison or jail should focus on building motivation for PrEP by employing strategies to increase awareness and knowledge about HIV risk and about PrEP as a prevention tool. Research on how best to engage people at risk for HIV during incarceration and around the time of release are needed. As ambivalence about PrEP use is addressed and interest increases, low-barrier access to PrEP will be critical for those motivated to use PrEP as an evidence-based HIV prevention tool.

## Supplementary Material

Supplementary Files

This is a list of supplementary files associated with this preprint. Click to download.

- SupplementalMaterialTopicGuide.docx

## Figures and Tables

**Table 1:** Characteristics of 15 Participants Interviewed

Age (years)	Mean (SD)
	41.8 (8.4)
	N (%)
Gender identity	
*Cisgender woman*	4 (26.7%)
*Cisgender man*	7 (46.7%)
*Transgender woman*	2 (13.3%)
*Transgender man*	1 (6.7%)
*Nonbinary*	1 (6.7%)
Race	
*Black or African American*	4 (26.7%)
*Native Hawaiian or Pacific Islander*	1 (6.7%)
*White*	7 (46.7%)
*More than one race*	2 (13.3%)
*Missing*	1 (6.7%)
Ethnicity	
*Hispanic or Latinx*	3 (20%)
*Not Hispanic or Latinx*	12 (80%)
Highest level of education	
*Did not graduate high school*	4 (26.7%)
*High school diploma or GED*	4 (26.7%)
*Some college, no degree*	4 (26.7%)
*College degree*	2 (13.3%)
*Missing*	1 (6.7%)
Most recently released from	
*Prison*	3 (20%)
*Jail*	12 (80%)

**Table 2:** Quotes about individual experiences and recommendations for others by COM-B and motivational PrEP cascade stage. Because there were relatively few quotes related to the action and maintenance stage, these are only included in the manuscript text.

Stage	Individual experiences	Recommendations for others
**Capability/Opportunity**	*“There's certain things that are super taboo in that world…talking about things that you're doing to better yourself and protect yourself…you just don’t do that. Because then…it’s like 'Well do you think you're better than us? What makes you special?'”* – White cisgender man, late 30s *“I was locked up for six years and before that there wasn’t a whole bunch of medical knowledge of how to prevent yourself from anything.”* – Black transgender man, early 30s *“There's not a lot of HIV educational pieces that are in prison or jail. And I don’t think people are aware that…I don’t think people take it seriously…”* – Pacific Islander nonbinary person, early 30s *“HIV, it’s a real thing. Now I hear it’s just a pill, and you can take it to where your numbers are so low, you technically don’t have it. But I'm sure the medication is super expensive.”* – White cisgender man, early 40s	*“If we had more free educational classes, I believe we'd have less of a problem with HIV going around as it does…So if we had the classes and put out flyers in or around those areas [libraries, recovery centers, colleges], I believe they would be gone to.”* – Black transgender man, early 30s *“I honestly believe that, when you first go to prison, when you first go to jail, there should be an option to be tested for HIV. If you have it, then you should be able to get the medication for it. And if you don’t have it, then you should be able to get PrEP. There should be a preventative, because who knows what’s going to happen? Because like I said, people get bored when they're in prison.”* – Black transgender man, early 30s *“A lot of people should be able to have open and easy access…a lot of them didn’t know about…resources that were out there. It really made me think…I don’t think a lot of people are putting the effort into making sure that they're well taken care for. So they'll come back…”* – Pacific Islander nonbinary person, early 30s
**Precontemplation** Low HIV transmission knowledge and awareness Low risk perception Ambivalence	*“I never used IV needles. So I wasn’t really, you know, whoring around or anything really too bad…I wasn’t really just with a whole bunch of women, especially skanky ones…I've had sexual relations with so many women, you know, unprotected. I have been tested before,…in 2010 or 11.”* – White cisgender man, early 40s *“I think [PrEP is] great for people who have like exposure for risks. And my friends [who] are gay take it. Mostly. I don’t know anyone who uses drugs who takes it just for drug use… I never really met anybody, over 10 years, that was a drug user that got [HIV] from it. I've heard about it but I've never met anyone.”* – White cisgender man, late 30s *“I don’t agree with putting something in your body…to fight off something that you don’t want to contract. I know that they have a preventative medicine called PrEP for HIV, but…I don’t get on anything like that. But I use protection, I use condoms and stuff like that.”* – White cisgender woman, early 40s *“I'm not sick. So I don’t need the medication.”* – Black cisgender man, early 40s	*“I think what is missing is the more personal piece, right? Because…I don’t know if we have similar experiences. Have you [ever been] incarcerated or had a similar [experience]? I think being more personal [would] really help with…a lot of disconnect and a lot of distrust.”* – Pacific Islander nonbinary person, early 30s *“What I'm saying is that it’s the prison system and the government or whatever, can access more people that the prisoners could go and speak to and let them know what’s going on, and they can try to access the opportunity to help them, it would be a lot less HIV.”* – Hispanic cisgender woman, early 50s
**Contemplation** HIV worry Increased knowledge and awareness about PrEP	*“I've always thought about diseases…I do drugs when I want to be intimate with people, mostly with women, from time to time. Sometimes, I don’t even worry about using protection, a condom or anything. When[ever] that happened, the next day I felt so bad about it that I was practically sick…I'm very afraid of HIV….since I was little I was told that it was certain death, it was dying, becoming very weak,…and not being able to have children.”* – Hispanic cisgender man, late 30s *“Well, I am concerned about HIV…I actually contracted hepatitis C in prison…there was no access to condoms, no access to clean needles. …I've always been a bit careful. And although I know I'm taking risks, I try to do what I call calculated stupid or calculated risks. Sort of just judging what I feel like is limited risk or low risk, and engaging in that at times, but trying to avoid what I feel is high risk. Since release from prison, I am interested in PrEP, or you know, having access to that, but I don’t even know how to do that. I don’t know that it’s accessible.”* – multiracial transgender woman, late 40s	*“If they have more knowledge about what they can and can’t use and what effects things have and don’t have, I believe that my community would be more knowledgeable about what to use for themselves.”* – Black transgender man, early 30s *“At least [information about PrEP] gives people the option to, if you're curious, research. And it’s something that you want to do, then do it if you're going to be active, do it”* – Pacific Islander nonbinary person, early 30s
**Preparation** Connection to resources Identification of PrEP barriers and facilitators	*“When I came here [local CBO post-release] and they gave me the [HIV] test, they saw me very worried. The person who gave me the test asked me a lot questions and I answered that I'm a drug addict, I'm an alcoholic and he told me, 'Apply for PrEP, because you're at risk, a lot of risk of having the disease.' Here, they helped me with the papers and all that”* – Hispanic cisgender man, late 30s	*“PrEP in the prisons probably would be a really good idea. I don’t know how you would pull that off. But yeah, that would be absolutely positive.”* – White transgender woman, late 50s

## Data Availability

The data supporting this study are sensitive qualitative records containing personal information from participants that could lead to their identification. They are intended solely for research purposes and will be accessible only to the named authors and any co-researchers who have signed a data use agreement. Access is restricted to secure, password-protected, encrypted storage. The data will not be shared publicly, used for commercial purposes, or disclosed without explicit written consent from the participants. Any request for access must be approved by the principal investigator and comply with institutional and ethical review board requirements.
